# Electrocardiographic characteristics of pediatric and adolescent football players

**DOI:** 10.1016/j.smhs.2023.12.004

**Published:** 2023-12-15

**Authors:** Maria Doumparatzi, Panagiota Sotiriou, Asterios Deligiannis, Evangelia Kouidi

**Affiliations:** Sports Medicine Laboratory, School of Physical Education and Sports Science, Aristotle University of Thessaloniki, Thermi, GR, 57001, Greece

**Keywords:** Exercise, Children, Adolescents, Electrocardiogram, Soccer

## Abstract

Electrocardiographic characteristics of children and adolescents present differences compared to adults. The aim of our work was to study electrocardiograms (ECGs) of football male players from childhood to late adolescence and examine if the ECG parameters are influenced by systematic exercise. One thousand fifty-four football players participated and formed four groups. Group A included 89 players aged 5–7 years, group B 353 players aged 8–11 years, group C consisted of 355 football players 12-15 yearsold and group D of 257 players with 16–18 years of age. All participants underwent preparticipation screening, including 12-lead surface ECG. Heart rate (HR), PR, RR, QRS, QT, QTc intervals, QT dispersion (QTdisp) and QRS axis were calculated. All ECGs were evaluated according to the current preparticipation cardiac screening guidelines, that refer to athletes aged 12–35 years and do not include pediatric players. Eleven percent of the participants presented an ECG finding. Group D obtained the lowest values of HR, QTc and the highest of PR, RR, QRS, QT intervals and QTdisp, whereas no differences in QRS axis were reported. Incomplete Right Bandle Branch Block (RBBB) was the most frequent ECG peculiarity, detected in 7.3% of the participants. Years of training were statistically significantly correlated to HR, PR, RR, QRS and QT intervals. In conclusion, guidelines for ECG interpretation of athletes in childhood, early and late adolescence are needed.

## Abbreviations list

Group A: Football players 5-7 years-old
Group B: Football players 8-11 years-old
Group C: Football players 12-15 years-old
Group D: Football players 16-18 years-old
WHO: World health organization
ECG: Electrocardiogram
RBBB: Right Bandle Branch Block
BSA: body surface area
HR: heart rate
bpm: beats per minute
h/wk: hours per week
QTdisp: QT dispersion
MRI: Magnetic resonance imaging

## 1 Introduction

Physical activity is essential for leading a healthy life. According to the latest guidelines by World Health Organization (WHO), 60 ​minutes (min) of moderate to vigorous exercise per day are needed in order to maintain a healthy status for children and adolescents aged 5–17 years; exercising more than 60 ​min per day is probably more beneficial.^1^ Nowadays, although many of them lead a sedentary lifestyle and do not meet the aforementioned exercise threshold,^2^ there is a growing number of others that engage to systematic physical activity in various sport disciplines, even from a very young age. As a result, preparticipation cardiac screening of athletes in the specific age group has become of utmost importance, so as to ensure safe practicing and to avoid disastrous events of sudden cardiac death.

The significance of cardiovascular preparticipation screening of young athletes has been established since the 1990s.^3^ At first, it aimed at evaluating professional and highly trained ones, but gradually became a prerequisite for all individuals that engage to systematic exercise in many countries worldwide. The inclusion of electro-cardiographic evaluation, apart from physical examination and personal/family history, has been a matter of debate for many years, mainly targeting the cost-effectiveness of the electrocardiogram (ECG). The major difference between the American and the European preparticipation cardiovascular screening guidelines is the inclusion of ECG in the latter.^4^

In 1980, Davignon et al. published their work that included 2 141 children's electrocardiographic evaluation.^5^ This was the first attempt to establish normal values for several pediatric electrocardiographic parameters. It was evident that children's and adolescents' ECGs, need to have special criteria for evaluation, as the heart and the conduction system go through developmental alterations. In 2001, Rijnbeek et al. provided new normal limits in pediatric electrocardiographic parameters after having studied 1 912 children^6^; more recently larger scale studies, mainly using digital ECG analysis, have tried to provide reference values. Yoshinaga et al. analyzed 56 ​753 children's and adolescents' ECGs in 2018^7^ and Bratincsák et al. provided normative values and *z*-scores for 102 parameters in 2020, after including 27 ​085 ECGs.^8^ It is evident, that the scientific interest and research on electrocardiographic adaptations with growing age is thriving. It is noteworthy, however, that the aforementioned studies have provided reference electrocardiographic values without including athletes.

Transition from childhood to early and late puberty is characterized by distinct electrocardiographic modifications, including lengthening of P and QRS wave duration, PR, QT intervals prolongation and on the contrary shortening of the QTc.^7^^,^^9^ Incomplete RBBB appears to be the most prevalent minor abnormality and complete RBBB the most frequent major one according to Santini et al.^9^ Repolarization peculiarities, including T wave inversion in chest leads V_1_–V_3_ are also common in ages of 12–16 years, however inverted T waves in chest leads V_5_–V_6_ should raise the suspicion of cardiac abnormality.^10^

Apart from age specific electrocardiographic alterations, the works that have appeared to date in literature, indicate that exercise-induced cardiac adaptations appear at a very early age. Pentikainen et al.^11^ have reported that electrocardiographic adaptations observed in adult athletes, can be detected in adolescent ones as well. McClean et al.^12^ have claimed that electrocardiographic alterations, both training and non-training related, are more common in pediatric athletes than non-athletes. Sinus bradycardia, first degree atrioventricular block, incomplete RBBB, voltage criteria for left ventricular hypertrophy and early repolarization are characteristic examples.^12^ Regarding structural cardiac exercise-induced adaptations, Bjerring et al.^13^ believe that children who continue to exercise through puberty evolve cardiac chamber dilatation, without myocardial hypertrophy. Works including early and late adolescent athletes of different non-Caucasian races have also been published^14^^,^^15^ all concluding in favor of the existence of training – related cardiac alterations in very young age. However, none of them included participants younger than 12 years. As a result, at present, guidelines applied for electrocardiographic evaluation of children and adolescents that exercise systematically are the ones used for individuals aged 12–35 years.^16^ Although they define which ECG findings are normal, which can be attributed to sports related cardiac adaptations and which are pathological, they contain no recommendations for children athletes.

Research interest regarding cardiac adaptations of pediatric and pubertal athletes is currently growing, aiming mainly to elucidate which cardiovascular characteristics can be attributed to maturation process, which to systematic exercise and which are caused by sinister heart conditions. The principal goal of our research was to study validated electrocardiographic parameters of athletes in childhood, early and late pubertal life period. Furthermore, we sought to evaluate the alterations that take place as age and training years accumulate and to recognize pathological findings which prompt further medical examinations.

## 2 Materials and methods

### 2.1 Subjects

The present observational study was conducted in Sports Medicine Laboratory of Aristotle University of Thessaloniki, Greece. All athletes were recruited through the national preparticipation screening program and were members of official football teams. Caucasian football male athletes aged between 5 and 18 years took part in the study and formed four groups, according to the national football academies’ classification. Group A included football players aged 5–7 years, group B 8-11 year-old players, group C footballers aged 12–15 years and group D 16–18 year-old players. According to Drezner et al.,^17^ systematic physical activity at least three times per week and 4 ​hours (h) accumulatively is required to exhibit training-related cardiac adaptations. The specific training threshold was a prerequisite for participating in the study. In addition, following a specific training routine and having the same coaching team throughout the study time period was a prerequisite among participants of the same group. Athletes who were on medication, were traumatized or ceased training during the study, were excluded.

The study protocol was approved by the Ethical Committee of Aristotle University of Thessaloniki (number of Approval No. ΕΗ-22/2020). All athletes who fulfilled the inclusion criteria and their parents/guardians provided informed consent before participating in the study.

### 2.2 Study protocol

Each participant or his parent/guardian filled in a detailed questionnaire regarding the athlete's personal and family medical history, the latter with emphasis to sudden cardiac death events. Additionally, anthropometric parameters were obtained. Consequently, physical examination took place including measurement of blood pressure, palpation of arteries in upper and lower extremities and auscultation of cardiac sounds. Then, a printed 12-lead surface ECG was obtained in supine position and quiet respiration with the use of Atria 3100 (Burdick, USA) ECG machine. The paper speed was 25 ​mm/s, the gain 1 mV/10 ​mm and the leads recorded were I, II, III, aVR, aVL, aVF and chest leads V_1_, V_2_, V_3_, V_4_, V_5_, V_6_, according to the standard procedure. All ECGs were obtained in morning hours between 09:00 a.m. and 12:00 p.m., in the same quiet environmental conditions. All participants refrained from exercise and heavy meals at least 12 ​h prior to the recording. Automatic analysis by the ECG machine software with the application of the Glasgow ECG Interpretation Algorithm, (University of Glasgow UK)^18^ took place. The following parameters were analyzed: heart rate (HR, beats per minute-bpm), the duration of PR, QRS, QT, RR intervals (ms), the duration of corrected QT interval that was calculated according to Bazett's formula (QTc in ms)^19^ and the axis of the QRS complex in degrees (°). In addition, the dispersion of the QT interval (QT disp in ms) i.e. the difference between the maximum and the minimum QT interval on surface ECG^20^ was calculated. The participants' physical examination and the evaluation of the digitally derived ECG parameters were conducted by an experienced sports cardiologist.

### 2.3 Statistical analysis

Statistical analysis of the obtained variables was conducted with the use of Statistical Package for Social Sciences (SPSS) version 27. Kolmogorov-Smirnov test was applied to test the normality of the distribution of quantitative parameters and consequently, parametric tests (ANOVA and Bonferroni post hoc test) were used for comparison of means of variables between groups. Correlations among indices were examined (Pearson's *r* index was calculated) and multiple regression analysis was also conducted. Results were considered statistically significant if *p* ​< ​0.05.

## 3 Results

A total of 1 054 football players participated in the study. Group A included 89 players, group B 353 players, group C 355 football players, and group D 57 players. Their anthropometric and training characteristics are exhibited in Table 1. All participants were normotensive. Blood pressure measurements were evaluated according to the previously published clinical practice guidelines.^21^ The accuracy of all automatically ECG-obtained parameters was confirmed by the sports cardiologist. The values of the electrocardiographic indices are shown in Table 2. Group D exhibited lower HR by 24.4% than group A (*p* ​< ​0.001), by 17.2% than group B (*p* ​< ​0.001) and by 10.4% than group C (*p* ​< ​0.001). Group D had the highest RR interval by 25.21% compared to group A (*p* ​< ​0.001) by 17.89% than group B (*p* ​< ​0.001) and by 11.36% than group C (*p* ​< ​0.001). The PR interval was higher in group D by 12.01% than group A, by 11.67% than B and by 6.68% than group C (*p* ​< ​0.001 for all comparisons) as was the QT interval by 10.75% (*p* ​< ​0.001) compared to group A, by 6.47% (*p* ​< ​0.001) to group B and by 3.98% (*p* ​< ​0.001) to group C. On the contrary, group D exhibited the lowest QTc interval by 3.7% than group A (*p* ​< ​0.001), by 2.7% than group B (*p* ​< ​0.01) and by 1.65% than C (*p* ​= ​0.01). Regarding QT disp, the index was lower in group B by 10% compared to group D (*p* ​< ​0.001) and by 6.45% to C (*p* ​< ​0.05). There was no statistically significant difference among groups with regards to the QRS axis.

**Table 1:** Anthropometric parameters, training age and weekly training hours of the participants (mean value ​± ​*SD*).

Variables	Group A 5–7 years old (*n* ​= ​89)	Group B **8**–**11 years old** (*n* ​= ​353)	Group C **12**–**15 years old** (*n* ​= ​355)	Group D **16**–**18 years old** (*n* ​= ​257)
Age (years)	6 ​± ​1.5	9.5 ​± ​2	13 ​± ​2.5	16.5 ​± ​1
Training age (years)	2.7 ​± ​1	3.8 ​± ​2	7.5 ​± ​1.9	9.6 ​± ​2.9
Training hours (h/wk)	4.3 ​± ​0.2	4.2 ​± ​0.1	4.4 ​± ​0.2	4.4 ​± ​0.3
Height (cm)	129.9 ​± ​6.6	140.8 ​± ​10.1	164.2 ​± ​11.4	177 ​± ​6.6
Weight (kg)	27.1 ​± ​4.2	35.4 ​± ​7.8	54.2 ​± ​11.9	68.6 ​± ​9.1
BSA (m^2^)	1 ​± ​0.1	1.2 ​± ​0.2	1.6 ​± ​0.2	1.8 ​± ​0.1

BSA: body surface area, h/wk: hours/week, Group A: Football players 5–7 years old, Group B: Football players 8–11 years old, Group C: Football players 12–15 years old, Group D: Football players 16–18 years old, *SD*: standard deviation.

**Table 2:** Values of electrocardiographic parameters among groups of participants (mean value ​± ​*SD*).

Variables	Group A 5–7 years old (*n* ​= ​89)	Group B 8–11 years old (*n* ​= ​353)	Group C 12–15 years old (*n* ​= ​355)	Group D 16–18 years old (*n* ​= ​257)
HR (bpm)	82.7 ​± ​12.5^a,b,c^	75.5 ​± ​11.5^b,c^	69.8 ​± ​11.9^c^	62.5 ​± ​12.6
PR (ms)	131.6 ​± ​15.5^b,c^	132.1 ​± ​16.1^b,c^	139.6 ​± ​18.3^c^	149.6 ​± ​21.4
QRS (ms)	80.6 ​± ​7.5^b,c^	82.1 ​± ​7.1^b,c^	87.8 ​± ​8.2^c^	94.4 ​± ​8.2
RR (ms)	736.8 ​± ​109^a,b,c^	809 ​± ​123^b,c^	873.3 ​± ​135^c^	985.3 ​± ​189.5
QT (ms)	355.2 ​± ​43.1^a,b,c^	372.2 ​± ​24.7^b,c^	382.2 ​± ​24.5^c^	398 ​± ​32.2
QTc (ms)	417.8 ​± ​20.7^d,c^	413.5 ​± ​22.7^c^	409.1 ​± ​23^e^	402.3 ​± ​25.5
QT disp. (ms)	46.2 ​± ​16.3	44.8 ​± ​13.8^d,c^	47.9 ​± ​15.3	49.8 ​± ​15.3
QRS Axis (°)	59.9 ​± ​25.1	63.4 ​± ​22.7	64.2 ​± ​24.7	65.7 ​± ​24.6

^a^*p* ​< ​0.001 vs group B, ^b^*p* ​< ​0.001 vs group C, ^c^*p* ​< ​0.001 vs group D, ^d^*p* ​< ​0.05 vs group C, ^e^*p* ​= ​0.01 vs group D, HR: heart rate, QTdisp: QT dispersion**,** bpm: beats per minute, Group A: Football players 5–7 years old, Group B: Football players 8–11 years old, Group C: Football players 12–15 years old, Group D: Football players 16–18 years old, *SD*: standard deviation.

The electrocardiographic characteristics of the participants are depicted in Table 3. It is noteworthy that 89% of the ECGs exhibited normal sinus rhythm, whereas 11% of them presented findings that needed further evaluation, for which the existing preparticipation screening guidelines were applied.

**Table 3:** Electrocardiographic characteristics of the participants.

Variables		Group Α 5–7 years old (*n* ​= ​89)	Group Β 8–11 years old (*n* ​= ​353)	Group C 12–15 years old (*n* ​= ​355)	Group D 16–18 years old (*n* ​= ​257)	Total (*n* ​= ​1 054)
Normal ECG	*n* (%)	79 (89.8 ​%)	325 (92.1 ​%)	316 (89.3 ​%)	206 (83.7 ​%)	926 (89.0 ​%)
Incomplete RBBB	*n* (%)	8 (9.1 ​%)	22 (6.2 ​%)	24 (6.8 ​%)	22 (8.9 ​%)	76 (7.3 ​%)
Sinus arrhythmia	*n* (%)	1 (1.1 ​%)	0 (0 ​%)	0 (0 ​%)	1 (0.4 ​%)	2 (0.2 ​%)
Atrial or ventricular extrasystoles	*n* (%)	0 (0 ​%)	1 (0.3 ​%)	1 (0.3 ​%)	4 (1.6 ​%)	6 (0.6 ​%)
(−) T in chest leads V1–V3	*n* (%)	0 (0 ​%)	2 (0.6 ​%)	6 (1.7 ​%)	0 (0 ​%)	8 (0.8 ​%)
R ​> ​S in chest lead V1	*n* (%)	0 (0 ​%)	1 (0.3 ​%)	0 (0 ​%)	0 (0 ​%)	1 (0.1 ​%)
(−) p in II, III, aVF	*n* (%)	0 (0 ​%)	1 (0.3 ​%)	0 (0 ​%)	1 (0.4 ​%)	2 (0.2 ​%)
RAD	*n* (%)	0 (0 ​%)	1 (0.3 ​%)	0 (0 ​%)	0 (0 ​%)	1 (0.1 ​%)
(−) T in III, aVF	*n* (%)	0 (0 ​%)	0 (0 ​%)	3 (0.8 ​%)	2 (0.8 ​%)	5 (0.5 ​%)
Repolarization abnormalities, biphasic T waves, (−) T in III	*n* (%)	0 (0 ​%)	0 (0 ​%)	1 (0.3 ​%)	4 (1.5 ​%)	5 (0.5 ​%)
(−) T waves in chest leads V_3_–V_5_, (−) T waves in lateral leads	*n* (%)	0 (0 ​%)	0 (0 ​%)	1 (0.3 ​%)	1 (0.4 ​%)	2 (0.2 ​%)
(−)T in chest leads V_4_–V_5_ (−)T in inferior and inferolateral leads	*n* (%)	0 (0 ​%)	0 (0 ​%)	1 (0.3 ​%)	1 (0.4 ​%)	2 (0.2 ​%)
Early repolarization pattern	*n* (%)	0 (0 ​%)	0 (0 ​%)	1 (0.3 ​%)	1 (0.4 ​%)	2 (0.2 ​%)
RBBB	*n* (%)	0 (0 ​%)	0 (0 ​%)	0 (0 ​%)	1 (0.4 ​%)	1 (0.1 ​%)
Tall peaked waves in chest leads V_2_–V_5_	*n* (%)	0 (0 ​%)	0 (0 ​%)	0 (0 ​%)	1 (0.4 ​%)	1 (0.1 ​%)
(−) T in inferior leads	*n* (%)	0 (0 ​%)	0 (0 ​%)	0 (0 ​%)	1 (0.4 ​%)	1 (0.1 ​%)

ECG: Electrocardiogram, RAD: Right Axis Deviation, RBBB: Right Bundle Brunch Block, Group A: Football players 5–7 years old, Group B: Football players 8–11 years old, Group C: Football players 12–15 years old, Group D: Football players 16–18 years old.

Correlations between the examined electrocardiographic indices and anthropometric parameters along with training age are shown in Table 4. Multiple statistically important correlations were revealed and multiple regression analysis led to the conclusion that age was independently positively correlated to HR, RR and QT intervals. The latter was also independently influenced by training age. In addition, QRS interval was statistically significantly affected by Body Surface Area (BSA) and weight.

**Table 4:** Correlations among indices.

	Age (years)	**BSA (m**^**2**^**)**	Height (cm)	Weight (kg)	Training age (years)	HR (bpm)	PR (ms)	QRS (ms)	RR (ms)	QT (ms)	QTc (ms)	QTdisp (ms)	QRS axis (°)
**Age (years)**	1												
**BSA (m**^**2**^**)**	0.790 (*p* ​< ​0.001)	1											
**Height (cm)**	0.008( NS)	0.021( NS)	1										
**Weight (kg)**	0.762 (*p* ​< ​0.001)	0.994 (*p* ​< ​0.001)	0.019 (NS)	1									
**Training age (years)**	0.689 (*p* ​< ​0.001)	0.739 (*p* ​< ​0.001)	0.034 (NS)	0.728 (*p* ​< ​0.001)	1								
**HR (bpm)**	−0.403 (*p* ​< ​0.001)	−0.455 (*p* ​< ​0.001)	−0.060 (NS)	−0.435 (*p* ​< ​0.001)	−0.386 (*p* ​< ​0.001)	1							
**PR (ms)**	0.286 (*p* ​= ​0.001)	0.391 (*p* ​< ​0.001)	0.018 (NS)	0.387 (*p* ​< ​0.001)	0.342 (*p* ​= ​0.001)	−0.256 (*p* ​< ​0.001)	1						
**QRS (ms)**	0.459 (*p* ​< ​0.001)	0.617 (*p* ​< ​0.001)	0.003 (NS)	0.614 (*p* ​< ​0.001)	0.462 (*p* ​< ​0.001)	−0.361 (*p* ​< ​0.001)	0.254 (*p* ​< ​0.001)	1					
**RR (ms)**	0.398 (*p* ​< ​0.001)	0.464 (*p* ​< ​0.001)	0.067 (NS)	0.458 (*p* ​< ​0.001)	0.410 (*p* ​= ​0.001)	−0.945 (*p* ​< ​0.001)	0.268 (*p* ​< ​0.001)	0.379 (*p* ​< ​0.001)	1				
**QT (ms)**	0.346 (*p* ​< ​0.001)	0.388 (*p* ​< ​0.001)	0.076 (NS)	0.378 (*p* ​< ​0.001)	0.376 (*p* ​= ​0.001)	−0.739 (*p* ​< ​0.001)	0.199 (NS)	0.337 (*p* ​< ​0.001)	0.730 (*p* ​< ​0.001)	1			
**QTc (ms)**	−0.148 (NS)	−0.197 (NS)	−0.04 (NS)	−0.198 (NS)	−0.129 (NS)	0.471 (*p* ​< ​0.001)	−0.164 (NS)	−0.129 (NS)	−0,467 (*p* ​< ​0.001)	−0.32 (*p* ​< ​0.001)	1		
**QTdisp (ms)**	0.105 (NS)	0.142 (NS)	0.045 (NS)	0.139( NS)	0.115 (NS)	−0.067 (NS)	0.043 (NS)	0.105 (NS)	0.062 (NS)	0.207 (NS)	0.184 (NS)	1	
**QRS axis (°)**	0.054 (NS)	0.062 (NS)	−0.15 (NS)	0.057( NS)	0.046 (NS)	−0.1 (NS)	0.087 (NS)	0.056 (NS)	0.112 (NS)	0.080 (NS)	−0.08 (NS)	0.101 (NS)	1

Pearson's *r* and the level of statistical significance are exhibited. BSA: body surface area, QT disp: QT dispersion, HR: Heart rate, bpm: beats per minute.

## 4 Discussion

Our observational study has obtained and evaluated electrocardiographic recordings from over 1 000 children, early and late adolescent athletes. Preparticipation screening is a prerequisite in Greece for training in organized official athletic groups and ECG is obligatory in Europe to complete the evaluation. The parameters that we chose to report are the ones more frequently applied in the literature.

With regards to the examined indices, we have noted that HR decreases with the progression of age, as the parameter was significantly lower in the older adolescents, compared to the other participants. This finding is in accordance with previous studies,^6^^,^^9^^,^^22^ which have documented analogous results. In the multi-cohort study of Harteveld et al.,^23^ HR decreased linearly from infancy to late childhood and reached a plateau thereafter until late adolescence, when a slight further decrease was reported. The researchers noted a significant effect of ageing on parasympathetic nervous system activity as well, which increased from infancy to late childhood in boys. This phenomenon can, in part, interpret the decline of HR as parasympathetic nervous activity is known to inversely affect the specific parameter. Apart from the effect of maturation on HR, systematic training plays an important role in its regulation, although reportedly less significant than in the case of adult athletes.^24^ Indeed, in our study, HR significantly correlated with training age. As expected, in inverse concordance with HR, RR interval increased with the progression from childhood to adolescence. Statistically significant positive correlation between RR, chronological and training age, indicates the interaction of both maturation process and exercise with the marker.

Regarding PR and QRS intervals in our sample, we agree with Rijnbeek et al.^6^ that PR interval and QRS increases from childhood years to puberty as older participants exhibited statistically significant longer PR and QRS duration compared to younger ones. However, in our study, PR interval was influenced more by training age, than chronological one, taking into account the correlation co-efficient. This delicate differentiation in our results can be explained by taking into consideration that the work of Rijnbeek et al.^6^ was not focused on athletic population. Current preparticipation screening guidelines, include the PR interval prolongation, among the normal exercise-induced rhythm adaptations.^16^ On the contrary, QRS duration, which was longer in our older adolescents as well, was mainly affected by weight, BSA, but also by age and training history. Previous publications in literature have identified the correlation between the index and somatometric characteristics.^22^^,^^25^ In the study of Cravarretta et al.,^22^ QRS duration was strongly correlated with weight and BSA, as were PR interval and QTc. We observed the effect of the parameter on PR interval and QRS as well. However, in our study, height did not affect the latter. Additionally, Mc Clean et al. have claimed that athletes older than 14 years present longer QRS duration compared to those younger,^12^ so the effect of both maturation process and systematic exercise cannot be ignored.

QRS axis, on the other hand, did not differ among groups and according to Rijnbeek et al.,^6^ this is anticipated. Although there is a rightward cardiac axis in the first months of life, because of right ventricular hypertrophy, the QRS axis modifications that take place after birth are limited to the first 3–6 months of life. With respect to QT interval, our analysis exhibited a significant effect of chronological age on the index, in agreement with the research of Benatar et al.,^26^ who have similarly reported increase of QT duration with the progression of age. According to the writers, the marker is also affected by testosterone levels which significantly increase in pubertal years in boys. We also observed an effect of training age, unlike the study of Turkman et al.,^27^ in which there was no difference in QT interval between athletes and non-athletes. With regards to QTc duration, Santini et al.,^9^ after having studied 24 ​062 Roman students aged 12–19 years, draw the conclusion that HR and QTc decrease as age progresses. This finding is in complete agreement with ours, as our older participants exhibited the lowest values of HR and QTc with statistically significant difference, compared to younger ones. However, no significant correlations between age and QTc duration values were found, probably because the parameter contains adjustment for heart rate. According to Triposkiadis et al.,^28^ comparing QTc interval between groups with statistically significant HR differences may be inappropriate. We also, in part, agree with Cravarretta et al.^22^ regarding the decrease of QTc interval in older participants, however we did not detect a significant effect of BSA on the index as they did. In addition, QT disp in our study was within normal limits, i.e. between 30 and 60 ​ms according to Malik et al.^29^ Although higher in participants aged 8–11 years compared to others, the specific parameter was unaffected by age, training, or BSA. Our findings are in part in agreement with the ones of Turkmen et al.,^27^ who did not observe any differences in QTdisp between training and sedentary students. Similarly, Akkus et al.^30^ did not detect any difference between adolescent swimmers and non-athletes in this parameter. However, Stoickov et al.^31^ in a recent study, suggest that athletes of static sports disciplines exhibit higher QTdisp values, compared to long distance runners. The fact that we have included young athletes of the same exercise habitus could be the main reason for not revealing any correlations with training in our study. Nevertheless, the specific index is of debated significance according to Malik et al.^29^

With regards to ECG special findings, Santini et al.^9^ noted that incomplete RBBB was the most frequent minor abnormality in their group of participants as it was in our sample, although it was observed in 7.3% of our athletes compared to the 15.24% in the Santini et al. study. It is noteworthy however, that our work recruited participants who were engaged to systematic physical training, whereas Santini et al. included a larger sample that also comprised non – athletes. Taking that into account, incomplete RBBB, cannot be characterized as an abnormality in our sample, but as a normal finding in athletic population, according to the existing preparticipation screening guidelines.^16^ Regarding other ECG peculiarities, the second more frequent one was the appearance of T wave inversion in chest leads V_1_–V_3_ which was found in 0.8% of the participants. Notably, the specific ECG characteristic disappeared in our older adolescents, complying with the preparticipation guidelines that consider the finding as normal, until the age of 16 (juvenile T waves).^16^ Right axis deviation and complete RBBB, which are considered borderline characteristics, were observed in 0.1% of the athletes. However, they did not lead to further evaluation, as they were isolated findings, with unremarkable clinical examination and without concomitant family history of inherited cardiac disease, or sudden cardiac death. Physiologically, in healthy athletic population, the specific findings express the adaptation of right heart chambers to systematic exercise, including right ventricular dilation and interventricular dyssynchrony.^32^ Indeed, complete RBBB was depicted in 2 athletes that were included in groups C and D, in which years of systematic training had already started to accumulate. On the contrary, T wave inversion in leads of the inferior and/or lateral myocardial segments prompted further testing, in accumulatively 1% of the athletes, i.e in 10 of them. They all underwent thorough cardiological evaluation, which included cardiopulmonary exercise test, holter monitoring, echocardiogram and magnetic resonance imaging (MRI). Two of the participants suffered from myocarditis and one was diagnosed with hypertrophic cardiomyopathy. The former ceased training for six months, according to the existing guidelines,^33^ underwent repetitive testing including MRI scanning and returned to practice thereafter. The latter was advised to abstain from competitive training and was referred to a specialized center for further follow up. The seven ones with non-pathological findings are under strict yearly cardiac evaluation. Finally, early repolarization pattern in inferolateral leads, which is considered a dynamic and benign pattern^34^ mainly attributed to exercise-induced increased vagal tone^17^ and tall peaked T waves^16^ were also found in our sample.

The rest of the findings included characteristics that can be observed in pediatric population irrespectively of athletic training, including extrasystoles, sinus arrhythmia and R ​> ​S in chest lead V1. According to Dickinson^10^ ventricular extrasystoles can be found in 0.2% – 2% of children and supraventricular ones in 15% – 40% of older ones. In our study, atrial and/or ventricular ectopic systoles were recorded in 0.6% of the participants, i.e in six of them. According to the existing guidelines, the appearance of two or more premature ventricular contractions per 10 ​s of ECG tracings are considered a possibly abnormal finding.^16^ Accordingly, two out of six participants exceeded this threshold and underwent further testing, which included echocardiogram, holter monitoring and cardiopulmonary exercise testing. The majority of the extrasystoles were monomorphic and in both athletes disappeared completely during exercise testing which, according to literature^10^ indicates their benign character. No sinister arrhythmias were depicted in holter tracings. Sinus arrhythmia, although of unprecise etiology, is common in all ages and benign in the vast majority of children. Regarding the R ​> ​S in chest lead V1 finding, it has been reported that children aged between 8 and 12 years occasionally present the specific feature.^12^ Accordingly, we reported one child in group B that possessed the characteristic.

Although, we cannot completely clarify which electrocardiographic characteristics can be attributed solely to exercise, it is noteworthy that training age presented a positive statistically significant correlation with QRS duration, PR, RR, QT intervals and a negative one with HR. Exercise inducibility of electrocardiographic features has to be taken into consideration, as athletes from early to late adolescence, who were also engaged longer in systematic physical training compared to younger ones, exhibited an electrocardiographic peculiarity in 10.7% and 16.3% respectively.

It is evident that children, preadolescent and adolescent athletes may present special ECG characteristics which, we believe, should be evaluated according to new guidelines. Recently, Ragazzoni et al.,^35^ have highlighted the need of the above as well and have proposed an algorithm of ECG evaluation, which appears easy to apply and will certainly be useful to the sports cardiology community. Large scale studies like ours, can be used to verify the proposed algorithm.

Finally, the fact that our observational study included exclusively male football players presents a limitation, so further studies are needed, in order to test if our results can be applied in female ones, or in children and adolescents engaged to different kinds of sports disciplines.

## 5 Conclusions

Athletes aged younger than 18 years form a distinct group of training individuals, that present characteristics of cardiac conducting system which can be attributed to maturation process and exercise adaptations. Electrocardiographic evaluation of athletes in childhood, early and late adolescence presents a challenging task that presupposes age-specific guidelines and clinical judgement. Large-scale studies specifying on very young systematically exercising athletes are needed, in order to ensure safe physical activity.

## Funding

This research received no external funding

## Submission statement

All authors have read and agreed with the manuscript content. While this manuscript is being reviewed for this journal, it will not be submitted elsewhere for review and publication.

## Ethical approval statement

Informed consent was obtained from each participant and his/her parents/guardians. The study was reviewed and approved by the Ethical Committee of Aristotle University of Thessaloniki no of Approval No. ΕΗ-22/2020.

## Authors’ contributions

M. Doumparatzi; Literature research, data collection, statistical analysis and revising of the manuscript.

P. Sotiriou: Literature research, data collection, statistical analysis, writing, editing and revising of the manuscript.

A. Deligiannis: Study conception and supervision, editing and revising of the manuscript.

E.Kouidi: Study conception and supervision, editing and revising of the manuscript.

## Conflict of interest

The authors declare that they have no known competing financial interests or personal relationships that could have appeared to influence the work reported in this paper.
